# B cell depletion with anti-CD20 mAb exacerbates anti-donor CD4^+^ T cell responses in highly sensitized transplant recipients

**DOI:** 10.1038/s41598-021-97748-9

**Published:** 2021-09-13

**Authors:** Asuka Tanaka, Kentaro Ide, Yuka Tanaka, Masahiro Ohira, Hiroyuki Tahara, Hideki Ohdan

**Affiliations:** Department of Gastroenterological and Transplant Surgery, Graduate School of Biomedical and Health Sciences, Hiroshima University, 1-2-3 Kasumi Minami-ku, Hiroshima, 734-8551 Japan

**Keywords:** Immunology, Medical research

## Abstract

Pretransplant desensitization with rituximab has been applied to preformed donor-specific anti-human leukocyte antigen antibody (DSA)-positive recipients for elimination of preformed DSA. We investigated the impact of pretransplant desensitization with rituximab on anti-donor T cell responses in DSA-positive transplant recipients. To monitor the patients’ immune status, mixed lymphocyte reaction (MLR) assays were performed before and after desensitization with rituximab. Two weeks after rituximab administration, the stimulation index (SI) of anti-donor CD4^+^ T cells was significantly higher in the DSA-positive recipients than in the DSA-negative recipients. To investigate the mechanisms of anti-donor hyper responses of CD4^+^ T cells after B cell depletion, highly sensitized mice models were injected with anti-CD20 mAb to eliminate B cells. Consistent with clinical observations, the SI values of anti-donor CD4^+^ T cells were significantly increased after anti-CD20 mAb injection in the sensitized mice models. Adding B cells isolated from untreated sensitized mice to MLR significantly inhibited the enhancement of anti-donor CD4^+^ T cell response. The depletion of the CD5^+^ B cell subset, which exclusively included IL-10-positive cells, from the additive B cells abrogated such inhibitory effects. These findings demonstrate that IL-10^+^ CD5^+^ B cells suppress the excessive response of anti-donor CD4^+^ T cells responses in sensitized recipients.

## Introduction

It has been reported that preformed DSAs are associated with detrimental effects in transplant recipients^1^. In kidney transplantation, preformed DSAs, regardless of whether they are HLA-class I or II, can trigger hyper acute, accelerated acute, and early acute antibody-mediated rejection (ABMR)^2, 3^. Acute and chronic ABMR are major factors of renal allograft dysfunction and loss^4^. In contrast to other types of organ transplantation, liver transplant (LT) recipients are considered resistant to ABMR caused by DSAs^5, 6^. However, recent studies suggest that liver allografts have a relative resistance to ABMR, but specific situations can override the liver’s natural resistance and defense mechanisms^7, 8^. In detail, preformed class I and/or II DSAs, detected by single-antigen bead analyses, with a mean fluorescence intensity (MFI) ≥ 5000, are independently correlated with a greater risk of death^9^. Furthermore, preformed class I DSA (MFI ≥ 5000) also disproportionately affects patients transplanted with a high calculated model for end-stage liver disease (MELD) score and those who received lower quality organs [donor risk index (DRI) > 1.5]^10^.

To eliminate the preformed DSAs, several desensitization protocols comprising plasmapheresis, splenectomy, intravenous immunoglobulins (IVIG), and/or anti-B cell immunosuppressant treatment in the recipients have been reported for successful transplant^11–13^. Among these, B cell depletion with the prophylactic use of rituximab has also been applied in preformed DSA-positive recipients^14, 15^. However, depletion of B cells may influence T cell allo-responses because B cells are effective antigen-presenting cells that can activate allo-specific T cells^16^. The cytokine release syndrome induced by rituximab may also enhance T cell activation^17^. Reportedly, rituximab can modulate the immunoresponse by secretion of IL-10 and macrophage inflammatory protein-1β^18^. However, few studies have clearly investigated whether immune modification after administration of rituximab promotes or inhibits T cell allo-response, even though T cells are also sensitized with alloantigens in DSA-positive sensitized patients.

We desensitized DSA-positive patients with rituximab and plasmapheresis, which was adopted from a protocol for ABO-blood type incompatible (ABO-I) transplant recipients^19^. We have recently shown that pretransplant desensitization with rituximab has a minimal effect on the alloreactive T cell responses by comparing ABO-I and ABO-compatible groups^20^. However, the previous study excluded DSA-positive recipients who might have preformed donor-reactive T cells. Hence, the objective of this study was to elucidate the impact of pretransplant desensitization with rituximab on the subsequent response of T cells to donor-antigens in DSA-positive transplant recipients. Moreover, a highly sensitized murine model was applied to investigate the mechanisms of the significant impacts of B cell depletion by injecting anti-CD20 monoclonal antibodies (mAbs) on anti-donor T cell responses.

## Results

### T cell immune responses before and after rituximab administration in desensitized patients

The baseline characteristics of the desensitized patients are listed in Table 1.

**Table 1:** Characteristics of ABO-incompatible and DSA-positive patients.

	ABO-incompatible (n = 45)	DSA-positive (n = 17)	*P* value
Recipient age, years, median (range)	53.0 (20–71)	61.0 (24–70)	0.50
**Gender**			< 0.01
Male	31	3	
Female	14	14	
**Organ**			0.63
Kidney	32	11	
Liver	13	6	
Donor age, years, median (range)	55.0 (23–78)	58.0 (28–74)	0.43
**Gender**			< 0.01
Male	17	14	
Female	28	3	
**Relationship**			0.98
Child	9	4	
Parent	8	3	
Sibling	2	1	
Spouse	26	9	
**HLA allele MM**			
A (0: 1: 2)	10: 19: 16	3: 9: 5	0.75
B (0: 1: 2)	3: 22: 20	0: 8: 9	0.51
DR (0: 1: 2)	7: 21: 17	0: 12: 5	0.12

A difference was considered significant if the p-value was <0.05.

**Figure 1:**
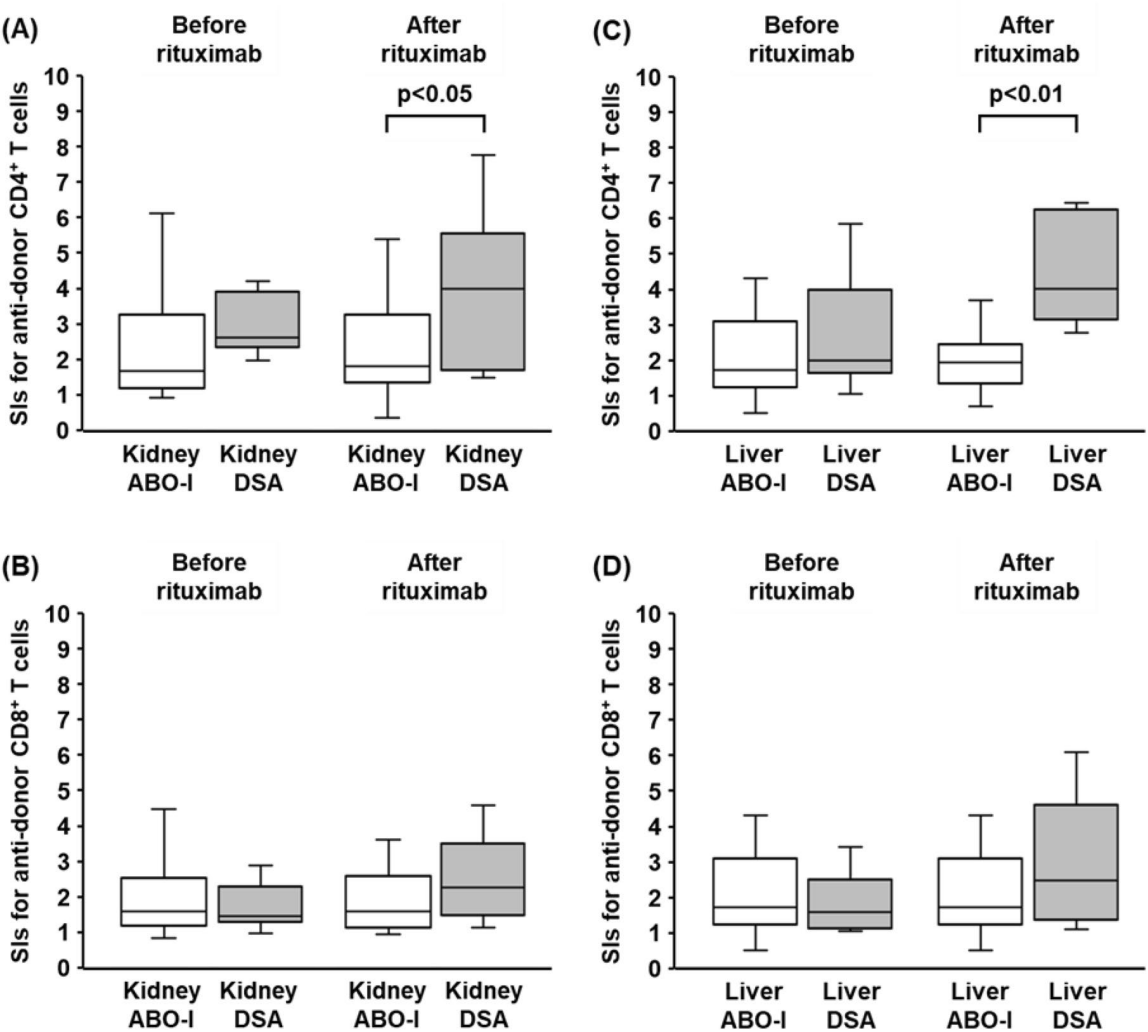
T cell immune responses before and after rituximab administration in desensitized patients. Sixty-two patients received pretransplant desensitization with rituximab. Of these, 17 patients were DSA-positive (kidney, n = 11; liver, n = 6) and the remaining 45 were DSA-negative ABO-I patients (kidney, n = 32; liver, n = 13). To evaluate the T cell immune status of recipients, CFSE-MLR assays were performed before rituximab administration and 2 weeks after rituximab administration (before transplantation). The stimulation index (SI) values of each CD4^+^ T cells (**A**, **C**) and CD8^+^ T cells (**B**, **D**) before and after rituximab are shown. White box, ABO-I group; gray box, DSA-positive group. Data are shown as median, 25th and 75th percentiles, and range. The Wilcoxon-Mann–Whitney test was used to evaluate differences between before and after desensitization.

Of these, 17 patients were DSA-positive (kidney, n = 11; liver, n = 6) and the remaining 45 were DSA-negative ABO-I patients (kidney, n = 32; liver, n = 13). Of the DSA-positive group, 9 patients were CDC cross match (XM)-positive (kidney, n = 5; liver, n = 4), and the remaining eight were CDC XM-negative flow cytometry crossmatch (FCXM)-positive patients (kidney, n = 6; liver, n = 2). In the DSA-positive group, there were more female recipients than males, and male donors than females. Similar to our previous report^20^, the proportion of peripheral blood IgM^+^ CD19^+^ B cells in all the recipients decreased below 0.1% at 2 weeks after administration of rituximab (data not shown). To evaluate the T cell immune status of recipients, we performed mixed lymphocyte reaction (MLR) assays using a carboxyfluorescein diacetate succinimidyl ester (CFSE)-labeling technique before rituximab administration and 2 weeks after rituximab administration (before transplantation). There were no significant differences in the SI values for the CD4^+^ T cell responses between the two groups before rituximab administration (Fig. 1A,C).

Of note, after rituximab administration, the SI values for the CD4^+^ T cell responses to donor stimulation were significantly higher in the DSA-positive group than those in the ABO-I group (*p* < 0.05, Fig. 1A,C). There were no significant differences in the SI values for the CD8^+^ T cell responses to donor between the two groups (Fig. 1B,D). After transplantation, kidney transplant recipients did not suffer from T cell-mediated rejection (TCMR), probably because they received either rabbit anti-thymocyte globulin (ATG) or basiliximab as induction therapy. In contrast, two out of five DSA-positive liver transplant recipients suffered from severe TCMR requiring rabbit ATG treatment.

### Kinetics of anti-allo Ab titers of a highly sensitized murine model

**Figure 2:**
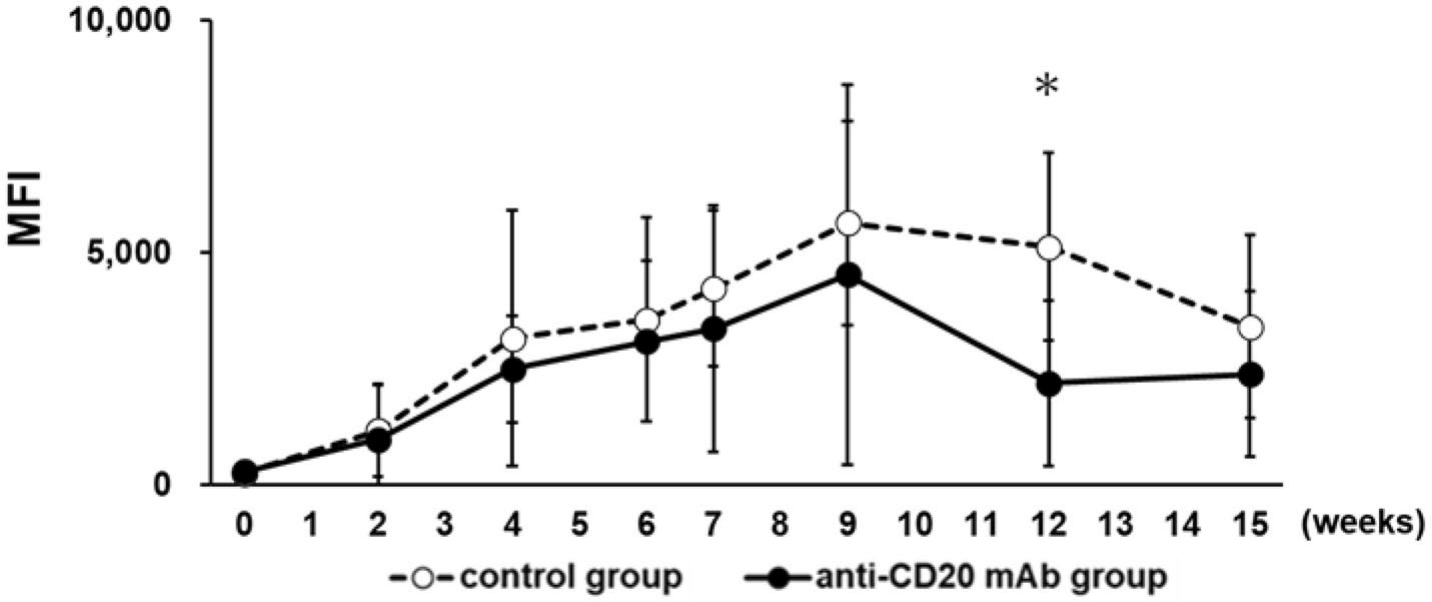
Kinetics of anti-donor Ab titers after allogenic skin transplantation. Donor C57BL/6 mouse tissues were transplanted twice onto the backs of the recipient Balb/c mice at 2-week intervals (weeks 0 and 2). Four weeks after the second skin transplantation (at week 6), recipient Balb/c mice were injected intravenously with 250 μg of the anti-CD20 murine IgG2b mAb (anti-CD20 mAb group, n = 5) or isotype control (control group, n = 5). The production of anti-C57BL/6 Abs in the sera of recipient Balb/c mice were evaluated with sera diluted 25-fold. Data are presented as MFI ± SEM; dotted line, control group; black line, anti-CD20 mAb group; *p < 0.05.

To investigate the mechanisms of anti-donor hyper responses of CD4^+^ T cells after B cell depletion, naïve Balb/c mice were transplanted with C57BL/6 skin grafts to prepare a highly sensitized model, and the sensitized mice were injected with anti-CD20 mAb to eliminate B cells. To confirm allo-sensitization by allogenic skin transplantation, the production of anti-C57BL/6 Abs in the sera of recipient Balb/c mice was serially evaluated. The levels of anti-C57BL/6 IgG titers gradually increased and reached a peak at nine weeks after the first skin transplantation (Figs. 2 and S1).

In the anti-CD20 mAb group (n = 5), unlike clinical desensitization therapy, plasma exchange was not performed in mouse experiments, and thus no dramatic decrease in anti-C57BL/6 IgG titers was observed. Instead, titers gradually decreased after injection of anti-CD20 mAb at week 6 after the first skin transplantation. The differences in anti-C57BL/6 IgG titers between the groups reached statistical significance at week 12 (*p* < 0.05).

### T cell immune responses after B cell depletion with anti-CD20 mAb in a highly sensitized murine model

**Figure 3:**
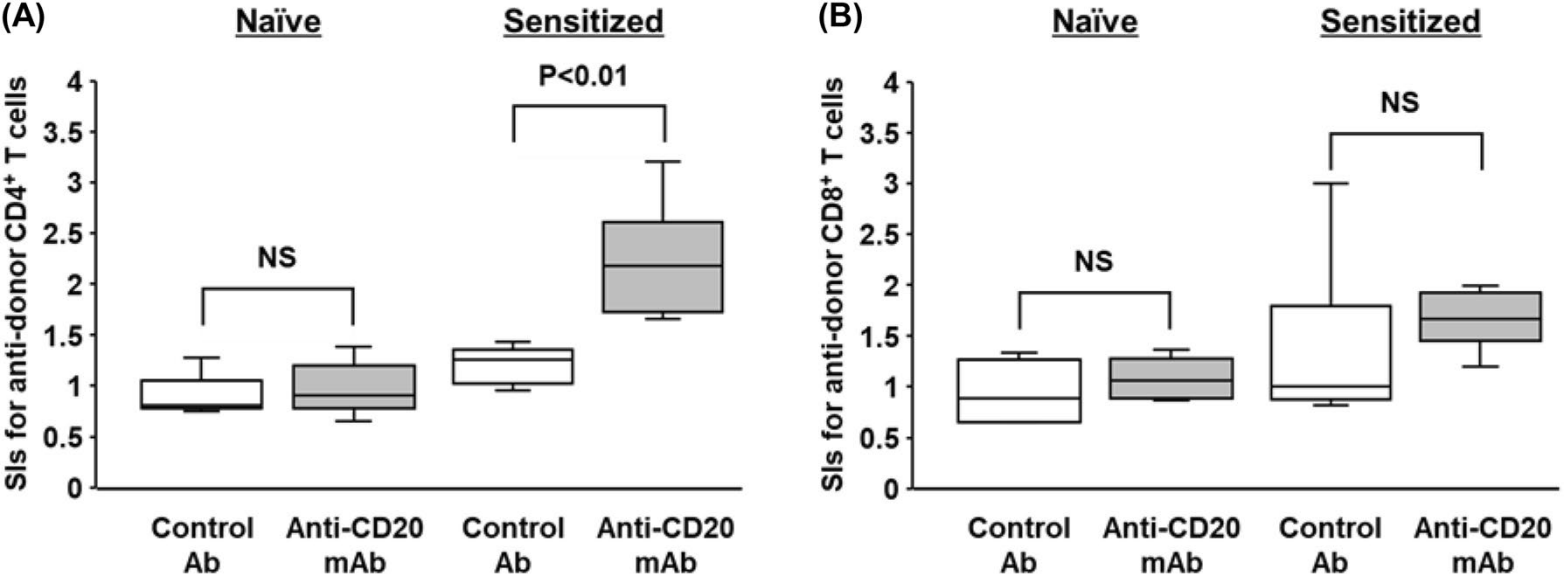
Anti-donor T cell immune responses after B cell depletion with anti-CD20 mAb in a murine model. One week after administration of either anti-CD20 mAb or isotype-matched control antibodies (control group) to either naïve mice or sensitized mice, CFSE-MLR assays were performed to evaluate anti-donor T cell responses. The SI values of each of the CD4^+^ T cell (**A**) and CD8^+^ T cell (**B**) for anti-donor responses are shown. White box, control group; gray box, anti-CD20 mAb group. Data from five independent experiments are shown as median, 25th and 75th percentiles, and range. The Wilcoxon-Mann–Whitney test was used to evaluate differences between the control and anti-CD20 mAb groups.

One week after administration of either anti-CD20 mAb (anti-CD20 mAb group, n = 6) or isotype-matched control Abs (control group, n = 6) to either naïve or sensitized mice, CFSE-MLR assays were performed to evaluate the T cell responses (at week 7). In naïve mice, there were no significant differences in SI values for the CD4^+^ and CD8^+^ T cell responses to donor and third party stimulation between the B cell depleting and control groups (*p* < 0.01, Figs. 3A,B, and S2).

However, consistent with the results of a human studies, the SI values for the CD4^+^ T cell responses to allo-stimulation in sensitized mice were significantly higher in the anti-CD20 mAb group than those in the control group (Figs. 3A and S2). There were no significant differences in SI values for the CD8^+^ T cell responses to allo-stimulation between the two groups (Figs. 3B and S2). These results indicate that B cell depletion in highly sensitized mice exacerbated CD4^+^ T cell responses to allo-stimulation, probably owing to elimination of B cell-mediated immune suppression.

### Evidence for the anti-donor hyper responses of CD4^+^ T cells induced by B cell depletion with anti-CD20 mAbs

**Figure 4:**
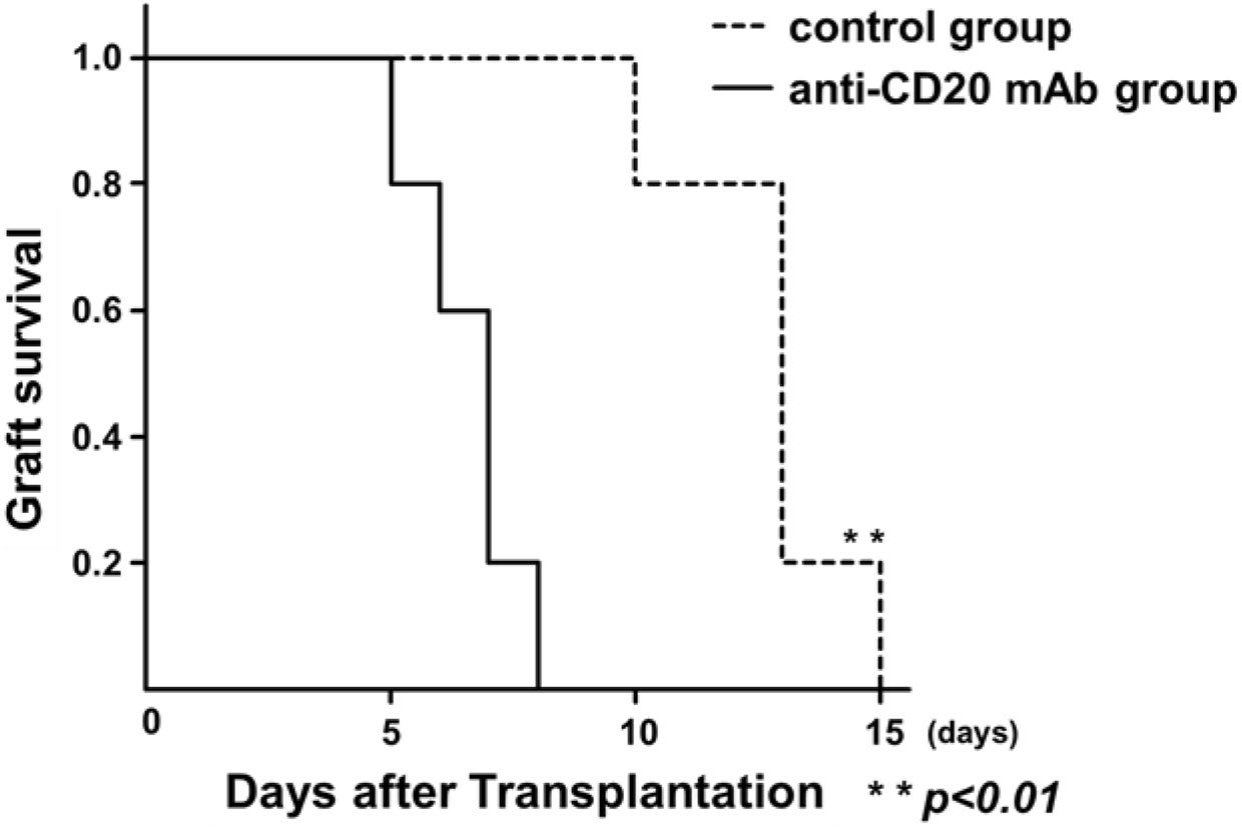
Effect of B cell depletion on allograft survival in sensitized mice. To confirm the anti-donor hyper-responses of CD4^+^ T cells induced by B cell depletion, the third time allogenic skin transplantation was performed at one week after B cell depletion with anti-CD20 mAb in the sensitized mice (n = 5), and the graft survival period was evaluated. The control group received isotype-matched control antibodies (control group, n = 5). Kaplan–Meier survival curves with log–rank statistics for graft survival are shown. Dotted line, control group; black line, anti-CD20 mAb group; **p < 0.01.

To investigate the impact of anti-donor hyper responses of CD4^+^ T cells induced by B cell depletion on the subsequently transplanted allografts cognate to the pre-engrafted skins, the third allogenic skin graft from C57BL/6 mice was transplanted at one week after B cell depletion with anti-CD20 mAb in the sensitized Balb/c mice (anti-CD20 mAb group, n = 5). The control group received isotype-matched control Abs (control group, n = 5). Despite the reduction in anti-C57BL/6 IgG titers, the third C57BL/6 graft survival period of the anti-CD20 mAb group of Balb/c recipients was significantly shorter than that of the control group (*p* < 0.01, Fig. 4). These results indicate that B cell depletion accelerated the acute cell-mediated rejection reaction, likely reflected by the anti-donor hyper responses of CD4^+^ T cells.

### Mechanisms for inhibitory effects of B cells on anti-donor CD4^+^ T cell responses

**Figure 5:**
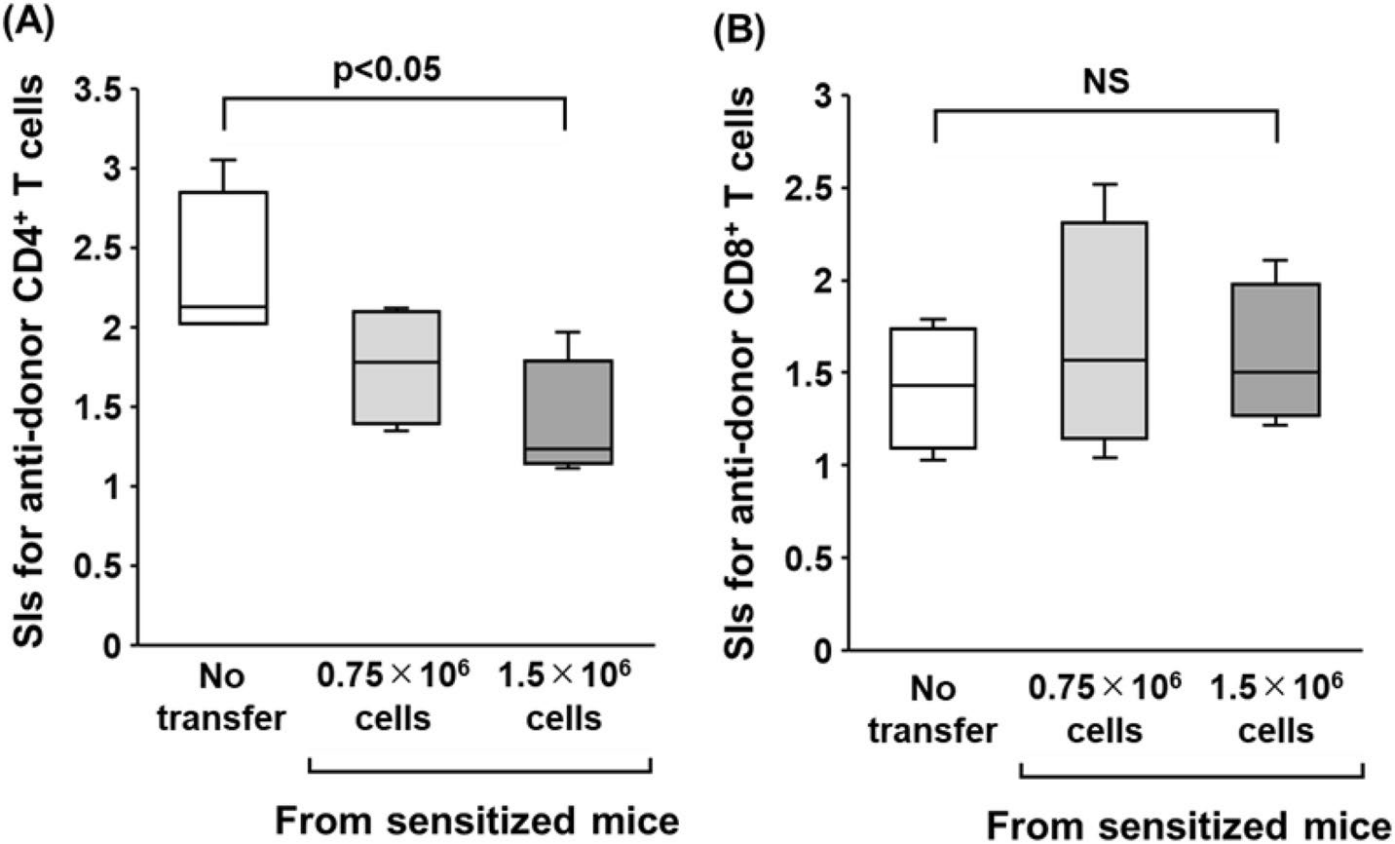
Evaluation of the dose effect of B cells from sensitized mice on anti-donor T-cell responses after B cell depletion with anti-CD20 mAb in a sensitized murine model. To investigate the inhibitory effects of B cells, various doses of B cells (0 × 10^6^, 0.75 × 10^6^, and 1.5 × 10^6^ cells/well) from sensitized mice were co-cultured in CFSE-MLR assays of desensitized mice, and the dose effect of B cells on anti-donor T-cell responses were evaluated. The SI values of each of the anti-donor CD4^+^ T cell (**A**) and CD8^+^ T cell (**B**) subsets in MLR are shown. Data from five independent experiments are shown as median, 25th and 75th percentiles, and range. The Wilcoxon-Mann–Whitney test was used to evaluate differences between the doses of B cells.

To investigate the inhibitory effects of B cells, various doses of splenic B cells (0 × 10^6^, 0.75 × 10^6^, and 1.5 × 10^6^ cells/well) were co-cultured in CFSE-MLR assays of desensitized mice, and the dose effect of B cells on anti-donor T cell responses was evaluated. Co-culture with 1.5 × 10^6^ B cells from sensitized mice significantly suppressed anti-donor CD4^+^T cell responses compared to those without B cell transfer (*p* < 0.05, Fig. 5A). Similar results were observed with B cells from naïve mice (Figure S3). There were no significant differences in SI values for the CD8^+^ T cell responses to donor stimulation regardless of sensitization (Figs. 5B and S3).

**Figure 6:**
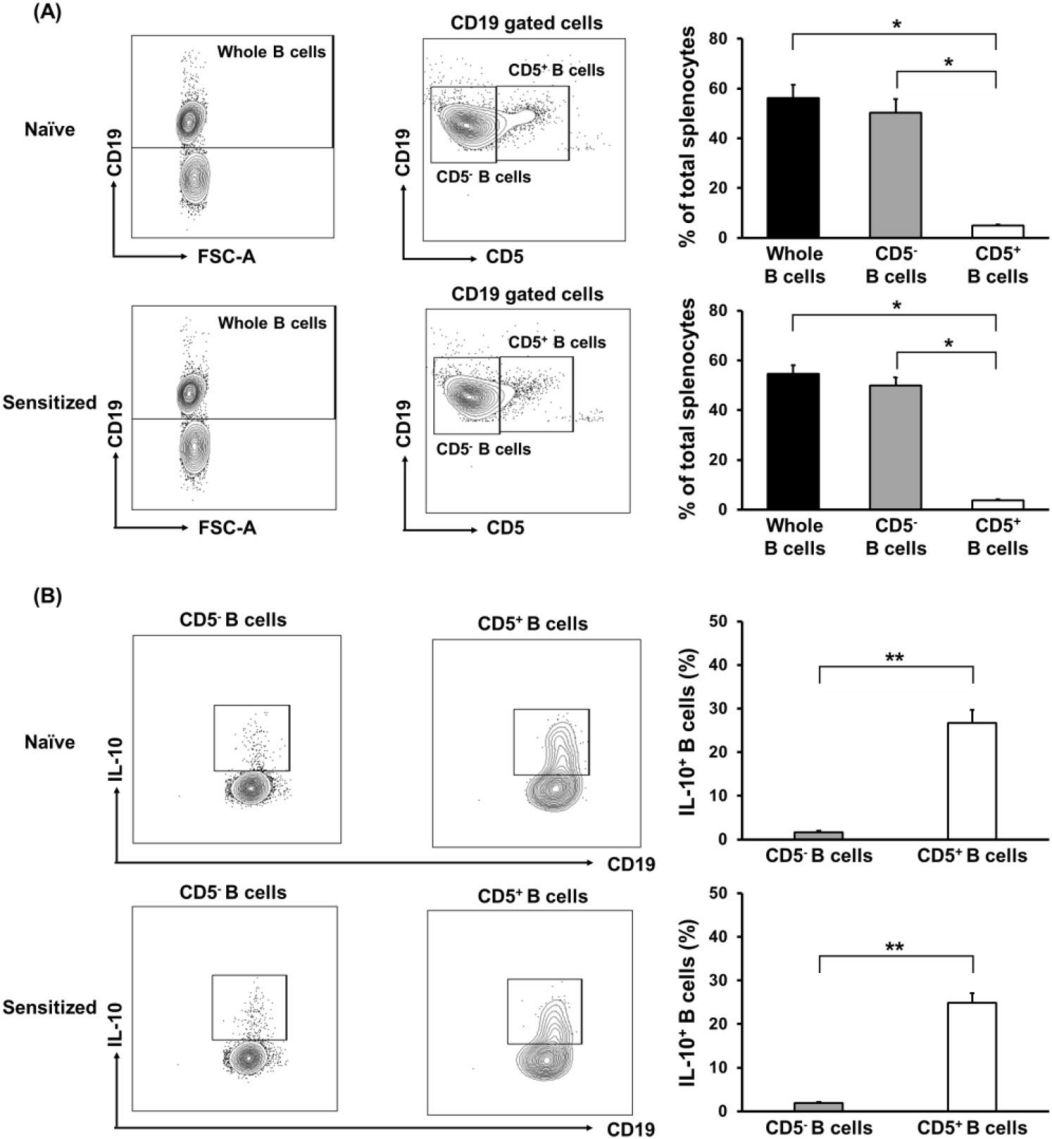
Analysis of CD5^+^ B cells and CD5^-^ B cells in naïve and sensitized mice splenocytes. (**A**) To investigate the phenotypic characteristic of B cells, splenocytes from either naïve or sensitized Balb/c mice were stained with anti-CD5 and anti-CD19 mAb. Representative FCM profiles are shown on the left column. The abundance ratios of whole B cells, CD5^-^ B cells, and CD5^+^ B cells from five independent experiments are shown on the right column. Upper column, naïve Balb/c mice; lower column, sensitized Balb/c mice, *p < 0.05. (**B**) To investigate the IL-10-positive B cells, splenocytes from either naïve or sensitized Balb/c mice were cultured with PMA, ionomycin, and monensin for 5 h and stained with anti-CD5 and anti-CD19 mAb. After permeabilization, the cells were stained with anti-IL-10 mAb. Representative FCM profiles are shown on the left column. The abundance ratios of IL-10-positive CD5^+^ B cells and CD5^-^ B cells from five independent experiments are shown on the right column. Data are presented as mean ± SEM. Upper column, naïve Balb/c mice; lower column, sensitized Balb/c mice, **p < 0.01.

We have previously demonstrated that CD5^+^ B-1 cells contain a distinguished subset, which potentially suppresses T cells responding to allostimulation^21^. Regardless of sensitization, CD5^+^ B cells were a small subpopulation of splenic B cells (Fig. 6A).

**Figure 7:**
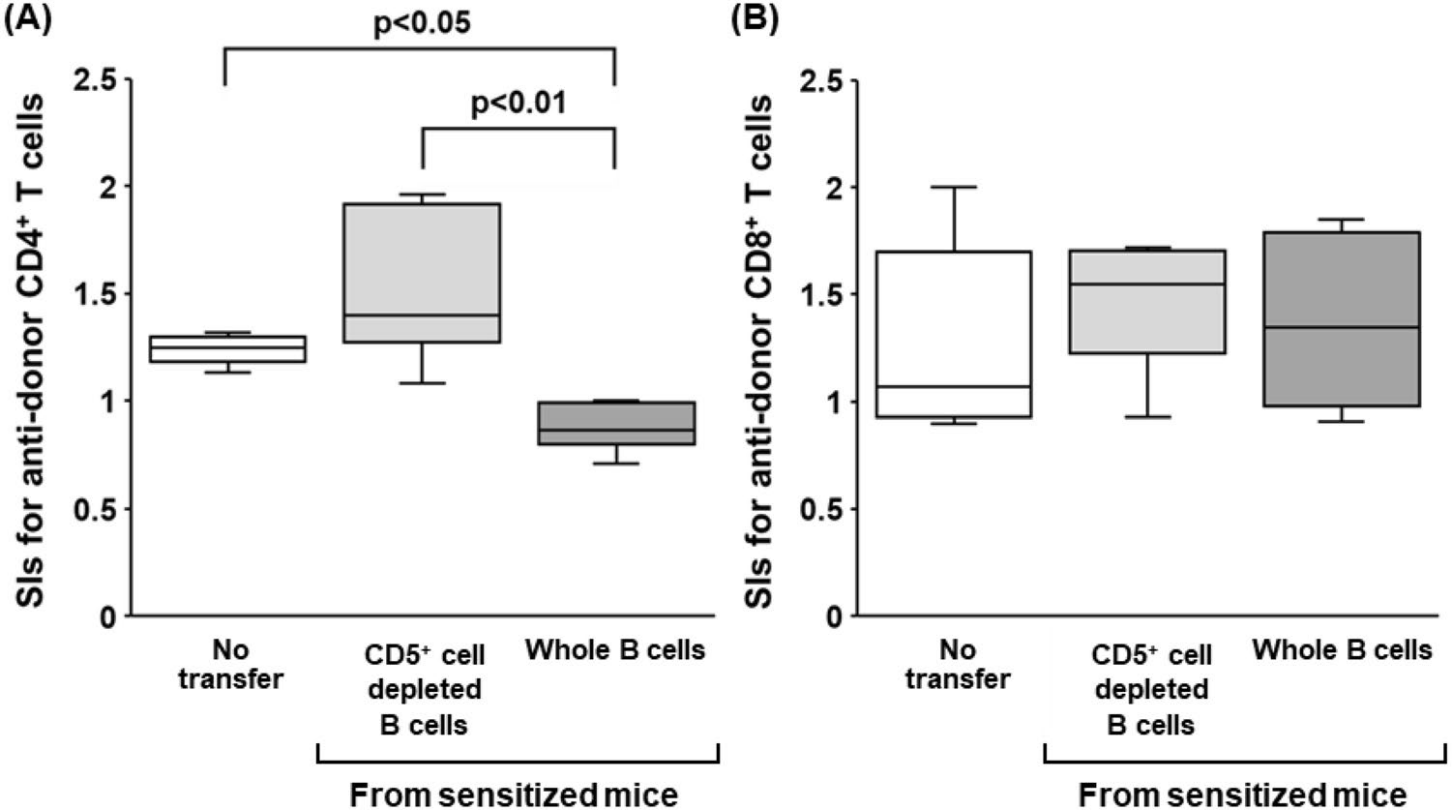
Effect of various B cell subsets from sensitized mice on anti-donor T-cell responses. To further investigate the inhibitory effects of B cells, either whole B cells or CD5^+^ B cell-depleted B cells from sensitized mice were co-cultured in CFSE-MLR assays of the desensitization group. The SI values of each of the anti-donor CD4^+^ T cell (**A**) and CD8^+^ T cell (**B**) subsets in MLR are shown. Data from five independent experiments are shown as median, 25th and 75th percentiles, and range. The Wilcoxon-Mann–Whitney test was used to evaluate differences between the groups.

Such a tiny B cell subset was enriched for IL-10-positive cells in both of naïve and sensitized mice (Fig. 6B). To further investigate the inhibitory effects of CD5^+^ B cells, either whole B cells or CD5^+^ cell-depleted B cells from either naïve or sensitized mice were co-cultured in CFSE-MLR assays of the desensitization group. Consistent with the results described above (Fig. 5), the addition of whole B cells from sensitized mice significantly suppressed anti-donor CD4^+^ T cell responses in the MLR (*p* < 0.05, Fig. 7A). Notably, CD5^+^ cell-depleted B cells significantly lost their ability to suppress CD4^+^ T cell allo-responses (Fig. 7A). A similar tendency was observed by adding either whole B cells or CD5^+^ cell-depleted B cells from naïve mice (Figure S4). There were no significant differences in SI values for the CD8^+^ T cell responses to donor stimulation regardless of B cell transfer (Fig. 7B). These results indicate that CD5^+^ B cells could inhibit the allo-responses of CD4^+^ T cells in allo-sensitized mice. Sensitization does not seem to significantly enhance the T cell inhibitory ability of CD5^+^ B cells, but the ability may be present in even naïve conditions.

## Discussion

B cells play an important role in the allogeneic stimulation of T cells. B cells not only constitute a complex system of antigen-presenting cells but may also regulate the immune response because they comprise separate subsets with different lineage affiliations, surface molecule expression, and biological functions. Benichou’s group has previously shown that B cell depletion enhances memory allo-responses by CD8^+^ T cells from OT-I TCR transgenic mice (recognition of MHC class I K^b^ bound to OVA peptide 254–267) sensitized with skin grafts of Act mOVA transgenic mice^22^. The same group recently demonstrated that B cell depletion impairs CD4^+^ allo-specific memory T cell responses after skin transplantation in transgenic Act mOVA mice in OT-II TCR transgenic mice (CD4^+^ T cells recognize MHC class II A^d^ bound to OVA peptide 323–335), indicating that B cell depletion exerts opposite effects on the development of CD4^+^ and CD8^+^ allo-specific memory T cells^23^. These studies underscore the complexity of the relationships between B and T cells in the generation and reactivation of different memory T cell subsets after transplantation. Because the Act-mOVA-to-OT-I/OT-II TCR transgenic mouse model used in these studies mimics CD4^+^ and CD8^+^ T cell responses induced via indirect allo-recognition, the influence of B cell depletion on T cells in recipients sensitized with fully allogeneic stimuli, which leads to both of direct and indirect allo-recognition, remains to be elucidated.

In the present study, we used a highly sensitized mouse model of a fully allogeneic combination and investigated the impact of B cell depletion with rituximab on donor-reactive T cells, which recognize alloantigens via either direct or indirect allo-recognition. We have previously shown that B cell depletion with rituximab has a minimal effect on alloreactive T cell responses in clinical settings of non-HLA sensitized patients^20^. The similar phenomenon was observed in this study of naïve mice, where no significant increase in the SI values for the CD4^+^ and CD8^+^ T cell responses to donor and third party stimulation by B cell depletion with anti-CD20 mAbs (Figs. 3 and S2). B cells require helper stimulatory signals from CD4^+^ T cells during the production of DSA^24^. Allo-sensitization may enhance the activation of allo-responsive T cells. However, the SI values of CD4^+^ and CD8^+^ T cells did not increase in MLR assays, which theoretically involve both direct and indirect recognition pathways^25^ even after allo-skin grafting. Paradoxically, the anti-donor CD4^+^ T cell responses were significantly increased after B cell depletion in the allo-sensitized recipients, both in clinical and mouse evaluations. Thus, depletion of B cells with anti-CD20 mAbs may hinder some inhibitory pathways, and thus the CD4^+^ T cells primed by either direct or indirect allo-recognition leads to hyper responsiveness.

B cells are typically characterized by their ability to produce Abs, function as secondary antigen-presenting cells, and produce various immunoregulatory cytokines. The regulatory B (Breg) cell population has been widely accepted as an important modulatory component of the immune system that suppresses inflammation^26, 27^. Breg cell populations are small under physiological conditions but expand substantially in both human patients and murine models of chronic inflammatory diseases, autoimmune diseases, infection, transplantation, and cancer^26–29^. We recently reported that PD-L1/PD-L2-expressing CD5^+^ B-1 cells have a suppressive effect on alloreactive CD4^+^ T cells in mice^21^. Such an immunoregulatory function of B-1 cells is mediated by direct cell–cell interactions and/or production of cytokines such as IL-10, resulting in the suppression of T cells. In the present study, we found that a small subset of CD5^+^ B cells exclusively contained IL-10-positive cells in the spleen of naïve and sensitized mice (Fig. 6). It is likely that IL-10-positive B cells, which express CD20 like conventional B cells, efficiently suppress the activation of alloreactive memory CD4^+^ T cells in sensitized mice. Depletion of B cells, including IL-10-positive B cells by anti-CD20 mAb, may result in the reactivation of alloreactive memory CD4^+^ T cells. If this hypothesis is true, selective depletion of CD5^-^ B cells, which include precursors of DSA-producing cells, may represent a promising strategy for desensitization that does not cause memory T cell reactivation. To address this possibility, additional studies employing either B cell-specific IL-10-deficient mice models^30^ or exogenous recombinant IL-10 supplementation are required.

In conclusion, our findings demonstrate that B cell depletion with rituximab exacerbates anti-donor CD4^+^ T cell responses in DSA-positive organ transplant recipients, and that it is probably associated with a high rate of TCMR events. It is possible that treatment with rituximab undesirably depletes IL-10-positive CD5^+^ B cells that regulate T cell responses to alloantigens. Further refinement of the desensitization protocol and novel agents are required to improve the outcomes of highly sensitized recipients.

## Materials and methods

### Study patients

The study was performed in accordance with the declaration of Helsinki and its amendments. This study was conducted with informed consent using a protocol approved by the institutional review board of the Hiroshima University Hospital (No. Hi-77). Between September 2007 and October 2019, 62 patients received pretransplant desensitization with rituximab, and were evaluated subsequent responses of T cells to alloantigens at Hiroshima University Hospital. Of these, 17 patients were DSA-positive (kidney, n = 11; liver, n = 6) and the remaining 45 were DSA-negative ABO-I patients (kidney, n = 32; liver, n = 13). The following information was collected at the time of transplantation: age, sex, primary disease, living donor relationship, human leukocyte antigen (HLA) mismatch, and results of the cross match (XM) test. Anti-HLA single-antigen reactivity was detected on a Luminex platform (LABScan 100 flow analyzer, Luminex, Austin, TX, USA) following the manufacturer’s protocol using LABScreen Single-Antigen assays. The results were recorded as the MFI. MFI values greater than 1000 were considered positive.

### Desensitization protocol and immunosuppressive regimen

This study was conducted with informed consent using a protocol approved by the institutional review board of the Hiroshima University Hospital (No. Hi-77). The patients were treated with a common desensitization regimen^20, 31^. First, a single dose of rituximab (375 mg/m^2^ body surface) was administered to patients. Subsequently, all subjects received calcineurin inhibitor (CNI) and mycophenolate mofetil (MMF) and underwent 0–5 sessions of plasmapheresis to decrease DSA titers. In kidney transplant recipient, either rabbit ATG or basiliximab was administered as induction therapy. The regimen after transplantation comprised CNI, MMF, and methylprednisolone with gradually tapering doses. The trough whole blood levels of CNI were the same between the DSA-positive and DSA-negative ABO-I patients (e.g., the trough whole blood levels of tacrolimus were maintained between 8 and 15 ng/mL for the first few postoperative weeks and between 5 and 10 ng/mL thereafter). The dose of MMF is 1–2 g administered in divides doses every 12 h.

### Immune monitoring by in vitro mixed lymphocyte reaction (MLR) assays

To evaluate the immune reactivity of patients, T cell responses to alloantigens were evaluated with MLR assays using an intracellular carboxyfluorescein diacetate succinimidyl ester (CFSE) labeling technique before rituximab administration and 2 weeks after rituximab administration (before transplantation) with the consent of the recipients and donors. After 5 days of incubation, the responder cells were harvested and subjected to multiparameter flow cytometric (FCM) analysis. CD4^+^ and CD8^+^ T cells were selected by gating and analyzed for CFSE fluorescence intensity. Stimulation indexes (SIs) were calculated as previously described^32, 33^.

### Mice

The animal studies conducted in this research were performed in compliance with the ARRIVE guidelines. C57BL/6 (H-2D^b^) and Balb/c (H-2D^d^) mice were purchased from CLEA (Osaka, Japan). C3H/HeNCrl (C3H; H-2D^k^) was purchased from Charles River Laboratories (Yokohama, Japan). Mice were housed in the animal facility of Hiroshima University, Japan, in a pathogen-free, micro-isolated environment. Female mice were used at an age of 7–12 weeks. Mice were euthanized by cervical dislocation after isoflurane inhalation, when indicated. All efforts were made to minimize the suffering of animals for the duration of their lives and during sacrifice^21^. This study was performed in strict accordance with the Guide for the Care and Use of Laboratory Animals and the local committee for animal experiments. The experimental protocol was approved by the Ethics Review Committee for Animal Experimentation of the Graduate School of Biomedical Sciences, Hiroshima University (Permit Number: A20-64-2). All animal experiments were performed according to the guidelines set out by the US National Institutes of Health (1996). This work was performed in part at the Research Facilities for Laboratory Animal Science, Natural Science Center for Basic Research and Development (N-BARD), Hiroshima University, Japan.

### Skin transplantation and B cell depletion with anti-CD20 mAbs

Donor C57BL/6 mouse tissues were transplanted twice onto the backs of the recipient Balb/c mice at 2-week intervals. Both donor and recipient mice were anesthetized with intraperitoneal injection of xylazine (5 mg/kg body weight) and ketamine (100 mg/kg body weight). Donor skin tissues were removed from the donor tails and trimmed into 5 mm × 5 mm strips. Two skin tissues of the same size were removed from the recipients’ backs and replaced with donor grafts. The skin grafts were covered with bandages for 5 days. Four weeks after the second skin transplantation (at week 6), recipient Balb/c mice were injected intravenously with 250 μg of anti-CD20 murine IgG2b mAb, SA271G2 (BioLegend, San Diego, CA) or rat IgG2b, κ isotype control. The third transplantation was performed in the same manner as described above one week after the administration of anti-CD20 murine IgG2b mAb or rat IgG2b, κ isotype control (at week 7).

### Cell preparation and flow cytometric analysis

Mononuclear cell suspensions from mouse spleen were prepared, and B cells purified by positive selection using CD19 MicroBeads (Miltenyi Biotec, San Diego, CA, USA) with an autoMACS Pro Separator (Miltenyi Biotec) according to the manufacturer’s instructions^21^. The purity of sorted cells was consistently > 95%. CD5^+^ B cells and CD5^-^ B cells were isolated using FACS Aria II(BD Biosciences). All cell cultures were performed in RPMI 1640 medium supplemented with 5% FBS, 2 mM L-glutamine, 100 U/mL penicillin–streptomycin, and 50 µM 2-ME. Mononuclear cells freshly isolated from splenocytes were fluorescently labeled with the following mAbs: anti-CD4- phycoerythrin (PE) (GK1.5), anti-CD8a-PE (53-6.7), anti-CD5-PE-Cy7 (53-7.3; BioLegend), anti-CD19-APC-Cy7 (1D3), and anti-CD5-PE (53-7.3)^21^. For intracellular cytokine staining, cells were stimulated in culture medium containing PMA (50 ng/mL; Sigma), ionomycin (500 ng/mL; Sigma), and monensin (2 mM; BD Pharmingen) in a 5% CO_2_ incubator at 37 °C for 5 h. After cell stimulation, for IL-10 detection, Fc receptors were blocked with mouse Fc receptor-specific mAb (2.4G2; BD Pharmingen) before cell surface staining, and then fixed and permeabilized with the Fixation/Permeabilization Solution Kit (BD Pharmingen) according to the manufacturer’s instructions. Permeabilized cells were stained with PE-conjugated anti-IL-10 mAb (JES5-16E3; BD Pharmingen). The antibodies used in this study were from BD Pharmingen, except for anti-CD5-PE-Cy7. The isotype-matched controls were used as follows: rat immunoglobulin G (IgG) 2a-κ-PE, rat IgG2b-κ-PE, rat IgG2a-κ-PE-Cy7, and rat IgG2a-κ-APC-Cy7. FCM assays were performed on a FACS Canto II (BD Biosciences). Nonspecific FcγR binding of labeled mAbs was blocked with anti-mouse CD16/32(2.4G2; BD Pharmingen, Hamburg, Germany). Dead cells were excluded from analysis using forward scatter and propidium iodide (PI; Sigma-Aldrich, St. Louis, MO, USA) or 7- amino-actinomycin D (7-AAD; BD Biosciences, Mountain View, CA, USA) staining. Results were analyzed using FlowJo software version 10.1 (Becton, Dickinson and Company, Franklin Lakes, NJ, USA).

### Antibody detection

Anti-C57BL/6 Abs were detected with indirect immunofluorescence staining of C57BL/6 mouse splenocytes using FCM assays. A total of 0.5 × 10^6^ splenocytes were incubated with 100 µL of serum for 1 h at 4 °C, and then for 30 min at 4 °C with FITC-conjugated anti-mouse IgG polyclonal Ab (11-4011; eBioscience, San Diego, CA), and APC-Cy7-conjugated anti-mouse CD19 (1D3; BD Pharmingen) to exclude nonspecific detection of IgM or IgG expressed by C57BL/6 splenic B cells. MFI values of CD19 negative cells were used to determine donor-specific Ab levels.

### CFSE-MLR assay

Splenocytes from Balb/c mice recipients at week 7 were labeled with 5 µM CFSE (Molecular Probes: Eugene, OR) as described previously^32^ and resuspended in culture medium for use as responder cells. Fractions of whole splenocytes were prepared from C57BL/6, Balb/c, and C3H mice. After each fraction was irradiated (30 Gy) for use as stimulator cells, 4 × 10^6^ stimulator cells and 4 × 10^6^ responder cells were co-cultured in 24-well flat-bottom plates at 37 °C in a 5% CO_2_ incubator in the dark. When indicated, 2 × 10^6^ stimulator cells and 1 × 10^6^ responder cells were placed in a 48-well flat-bottom plate, and either 1.5 × 10^6^ whole B cells or CD5^+^ B cell-depleted B cells were added. After 4 days of incubation, the responder cells were harvested and analyzed using FCM. CD4^+^ and CD8^+^ T cells were selected by gating and analyzed for the intensity of CFSE fluorescence. SIs were calculated as previously described^32, 33^.

### Statistical analysis

Statistical analyses were performed using JMP statistical software, version 15 (SAS Institute, Cary, NC). The Chi-square test or Fisher’s exact test were used for the comparison of categorical variables and the Student’s *t*-test or the Mann–Whitney *U*-test for continuous variables. Differences in SI values before and after desensitization were tested using Wilcoxon signed rank test. Comparisons between groups were made using analysis of variance (ANOVA), and significant differences were examined with Tukey–Kramer multiple comparison post hoc test. Survival rates of the skin allografts were calculated using Kaplan–Meier/log-rank test. P-values below 0.05 were considered statistically significant.

## Supplementary Information

Supplementary Figures.
