# Supplementary material for: B cell depletion with anti-CD20 mAb exacerbates anti-donor CD4+ T cell responses in highly sensitized transplant recipients
Source: Sci Rep. 2021 Sep 13;11:18180. doi: 10.1038/s41598-021-97748-9 (PMC8437972; doi:10.1038/s41598-021-97748-9)
Supplement: Supplementary file 1 — Supplementary Figures. [file 41598_2021_97748_MOESM1_ESM.pdf]

Supplemental Figure legend

Figure S1. Kinetics of anti-donor Ab titers after allogenic skin transplantation.

Donor C57BL/6 mouse tissues were transplanted twice onto the backs of recipient Balb/c mice at 2-week intervals (weeks 0 and 2). Four weeks after the second skin transplantation (at week 6), recipient Balb/c mice were injected intravenously with 250 μg of the anti-CD20 murine IgG2b mAb (anti-CD20 mAb group, n = 5) or isotype control (control group, n = 5). The production of anti-C57BL/6 Abs in the sera of recipient Balb/c mice were evaluated with FCM assays. Sera were diluted to 1-fold (A) and 5-fold (B) concentrations. Data are presented as MFI ± SEM; dotted line, control group; black line, anti-CD20 mAb group; *$p < 0.05$.

Figure S2. Anti-third party T cell immune responses after B cell depletion with anti-CD20 mAb in a murine model.

The SI values of each of the $CD4^+$ T cell (A) and $CD8^+$ T cell (B) for anti-third party responses are shown. White box, control group; gray box, anti-CD20 mAb group. Data are shown as median, 25th and 75th percentiles, and range. The Wilcoxon-Mann-Whitney test was used to evaluate differences between the control and anti-CD20 mAb groups.

Figure S3. Evaluation of the dose effect of B cells from naïve mice on anti-donor T cell responses after B cell depletion with anti-CD20 mAb in a sensitized murine model.

To investigate the inhibitory effects of B cells, various doses of B cells (0×10^6, 0.75×10^6, and 1.5×10^6 cells/well) from naïve mice were co-cultured in CFSE-MLR assays of desensitized mice (n = 5), and the dose effect of B cells on anti-donor T cell responses were

evaluated. The SI values of each of the anti-donor $CD4^+$ T cell (A) and $CD8^+$ T cell (B) subsets in MLR are shown. Data are shown as median, 25th and 75th percentiles, and range. The Wilcoxon-Mann-Whitney test was used to evaluate differences between doses of B cells.

Figure S4. Effect of various B cell subsets from naïve mice on anti-donor T cell responses. To further investigate the inhibitory effects of B cells, either whole B cells or $CD5^+$ B cell-depleted B cells from naïve mice (n = 6) were co-cultured in CFSE-MLR assays of the desensitization group. The SI values of each of the anti-donor $CD4^+$ T cell (A) and $CD8^+$ T cell (B) subsets in MLR are shown. Data are shown as median, 25th and 75th percentiles, and range. The Wilcoxon-Mann-Whitney test was used to evaluate differences between the groups.

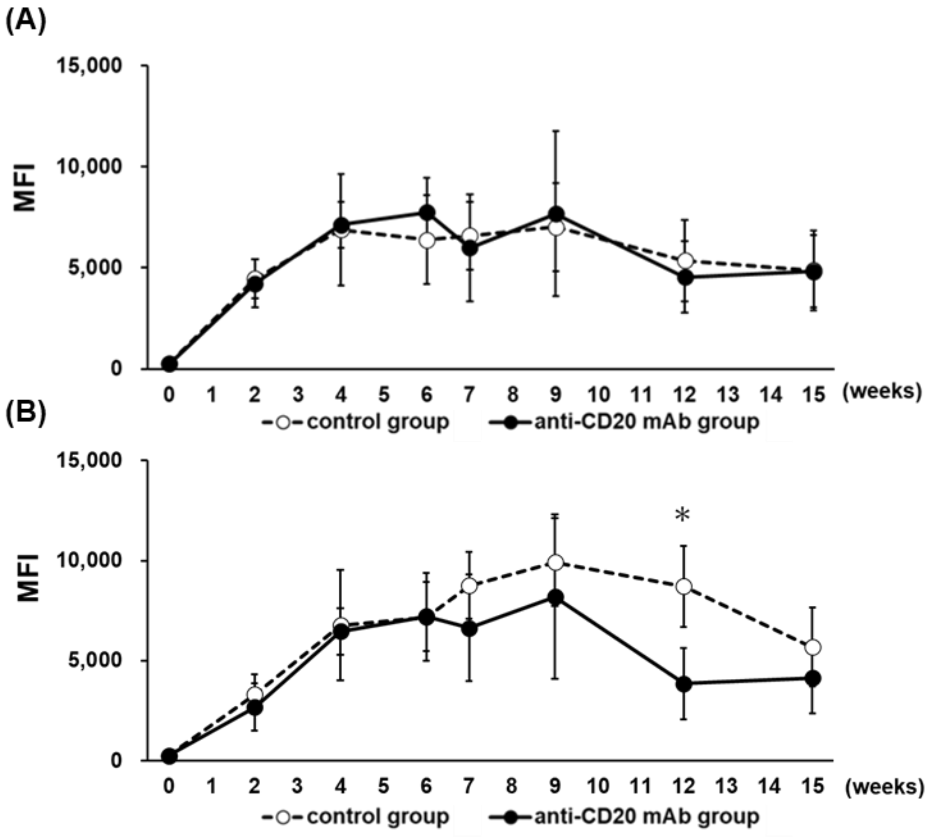

Supplemental Figure

(Figure S1)

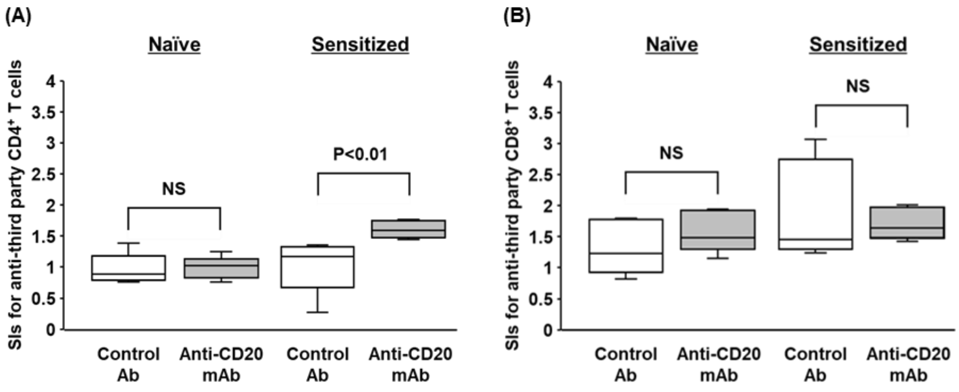

Supplemental Figure

(Figure S2)

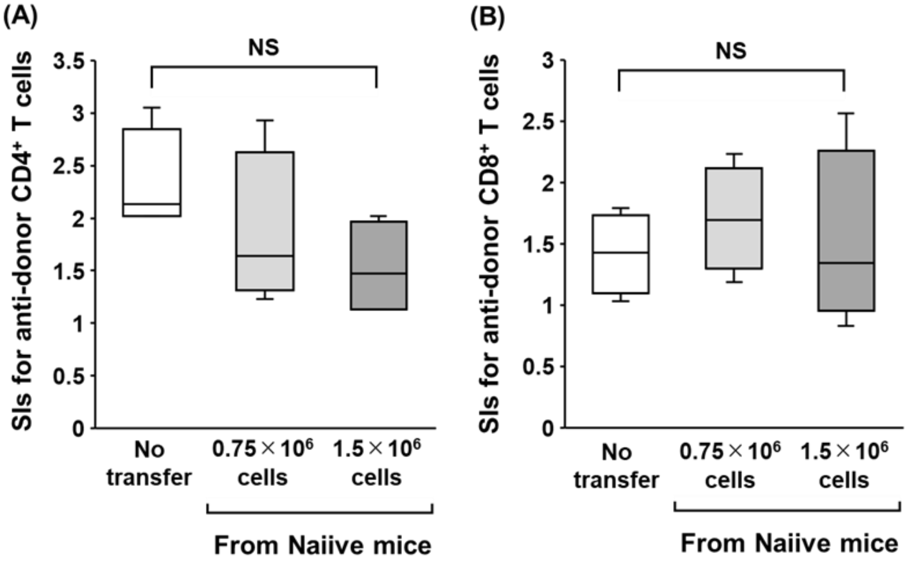

Supplemental Figure

(Figure S3)

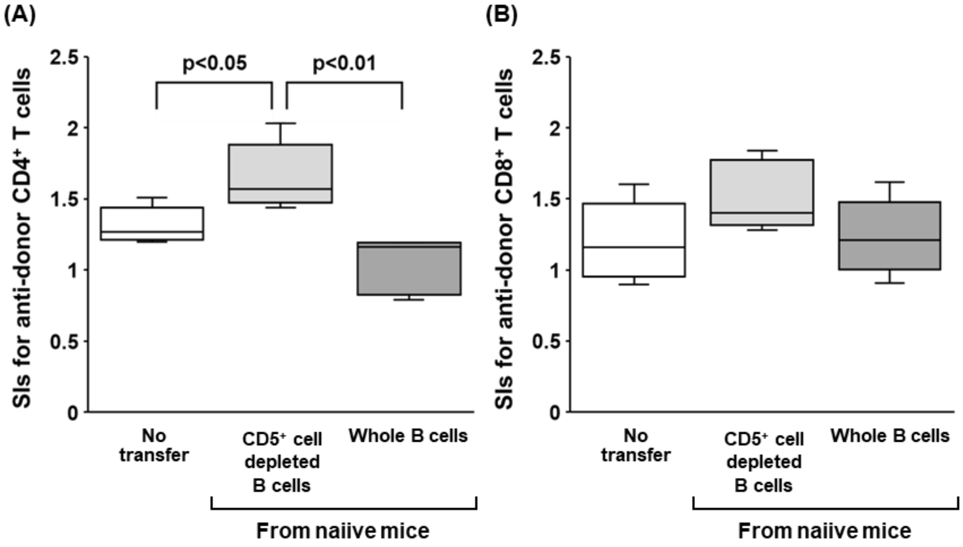

Supplemental Figure

(Figure S4)
